# Unilateral Tonsillar Swelling in a 52-Year-Old Heterosexual Greek Male With Secondary Syphilis: A Case Report Emphasizing the Main Histopathological Differential Diagnosis

**DOI:** 10.7759/cureus.81407

**Published:** 2025-03-29

**Authors:** Menelaos G Samaras, Evangelos Panagoulis, Christakis Kotanidis, Sotirios Karamagkiolas, Periklis G Foukas

**Affiliations:** 1 Second Department of Pathology, National and Kapodistrian University of Athens, "Attikon" University Hospital, Athens, GRC; 2 Second Otolaryngology Department, National and Kapodistrian University of Athens, "Attikon" University Hospital, Athens, GRC; 3 Second Department of Pathology, National and Kapodistrian University of Athens, “Attikon” University Hospital, Athens, GRC

**Keywords:** histopathology of syphilis, oropharyngeal malignancies mimickers, oropharyngeal syphilis, sexually transmitted diseases, unilateral tonsillar swelling

## Abstract

This case report describes the clinical presentation, diagnosis, and management of a 52-year-old Greek male with unilateral tonsillar swelling and concomitant syphilis, emphasizing the key histopathological differentials. The patient presented with a longstanding unilateral right tonsillar enlargement, and further evaluation revealed syphilis as the underlying disease. The management included intramuscular penicillin therapy and close follow-up with dermatology and otolaryngology specialists. This report aims to raise awareness among healthcare providers, particularly pathologists, about considering tonsillar syphilis in the differential diagnosis of tonsillar abnormalities, especially in patients at increased risk for syphilis infection.

## Introduction

Syphilis is a sexually transmitted infection caused by the spirochete bacterium *Treponema pallidum*. It is a global health concern [1]. The World Health Organization (WHO) estimates that in 2022, approximately 8 million adults aged 15-49 acquired syphilis [2]. Overall in Europe and particularly in Greece the incidence of syphilis has significantly increased from 2014 to 2023 in both sexes, especially in men [3]. Although syphilis primarily affects the genital region, it can also manifest in extragenital sites, such as the oral cavity. Syphilis progresses through primary, secondary, and tertiary stages, with oropharyngeal involvement typically occurring in secondary syphilis. Understanding this progression is crucial for recognizing atypical presentations, like tonsillar syphilis [2]. Tonsillar syphilis involves syphilitic lesions on the tonsils and can present with diverse clinical manifestations. It is rare, with only a few cases reported in the literature [4]. Risk factors include high-risk sexual behavior, HIV co-infection, and immunosuppression [1]. Here, we present a unique case of a 52-year-old male who exhibited unilateral tonsillar swelling, a rare manifestation of primary syphilis. This report highlights key histopathological differentials to aid in accurate diagnosis and prevent misclassification of tonsillar syphilis.

## Case presentation

**Figure 1:**
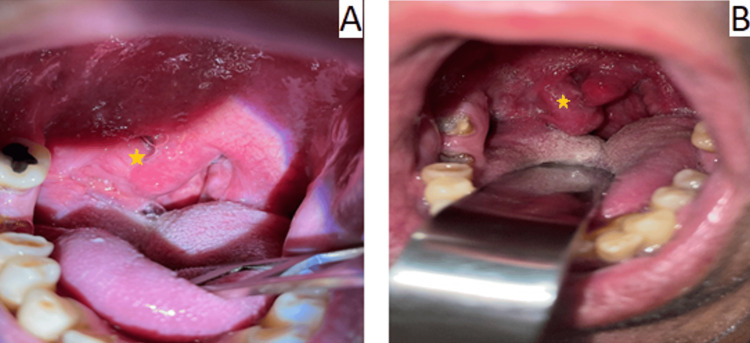
Clinical examination characteristics. A) Initial presentation of the patient. The star highlights the unilateral, asymmetrical swelling of the right palatine tonsil. B) After treatment with penicillin. A noticeable reduction in the size of the tonsil is observed.

**Figure 2:**
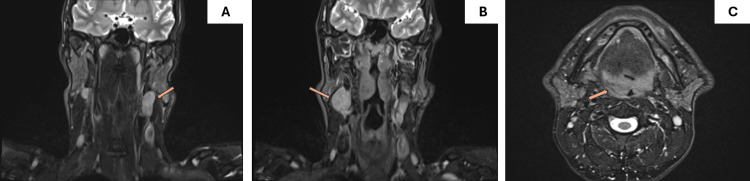
T2-weighted MRI scan without contrast enhancement. A) Coronal plane: Enlargement of left cervical lymph nodes, the largest of which measures 3.3 × 2.2 × 2.6 cm (arrow). B) Coronal plane: Enlargement of right cervical lymph nodes, the largest of which measured 3.8 × 2.2 × 1.9 cm (arrow). C) Axial plane: Asymmetric enlargement of the right palatine tonsil (arrow).

A 52-year-old Greek heterosexual male presented to our hospital’s outpatient clinic with a gradual onset of unilateral right tonsillar swelling, but could not recall its exact onset (Figure 1). Notably, he denied experiencing pain associated with the swelling. His medical history was unremarkable, except for a penile ulcer that developed approximately one year prior. He reported no history of surgeries, medication use, or known allergies. Upon examination, he exhibited unilateral right tonsillar swelling with no other oropharyngeal abnormalities aside from poor dental hygiene. Nasopharyngeal examination revealed no abnormalities, and laryngoscopy demonstrated normal findings. Evaluation of lymph nodes revealed palpable, swollen lymph nodes bilaterally. Neurological and ophthalmological assessments revealed no pathological findings. Pure-tone audiometry (PTA) demonstrated a normal hearing level for the patient’s age. Magnetic resonance imaging (MRI) revealed an asymmetric right tonsillar swelling and multiple swollen cervical lymph nodes bilaterally (left: 3.3 × 2.2 × 2.6 cm and right: 3.9 × 2.2 × 1.9 cm) (Figure 2). A tonsillar biopsy was performed to rule out malignancy.

**Figure 3:**
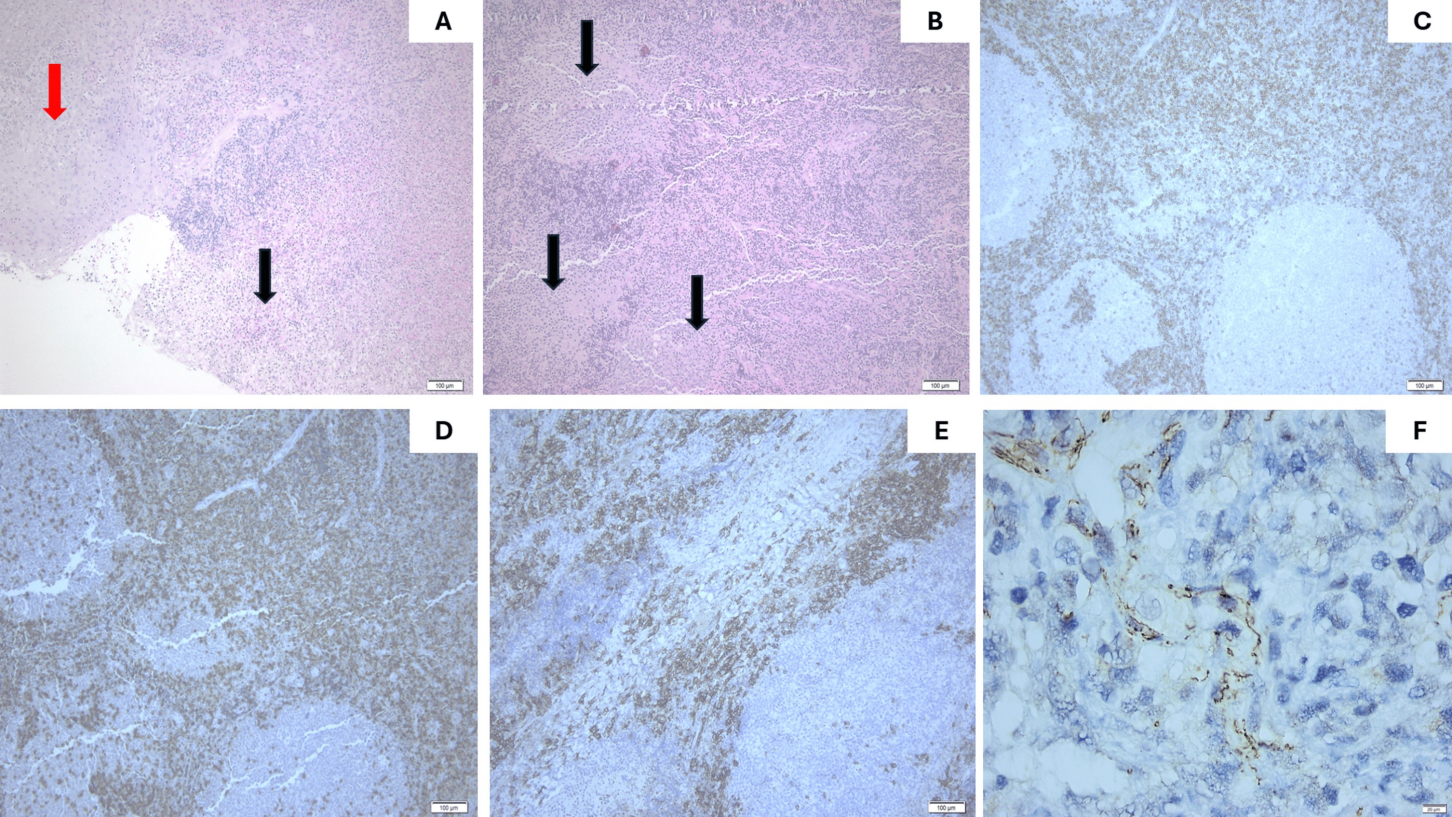
Histopathological features. On low-power magnification an ulcerative focus (black arrow) with deep extension of the fibrinopurulent exudate into the underlying stroma, adjacent to mature squamous epithelium (red arrow) is readily observed (A), Hematoxylin and Eosin stain, 100×). The underlying stroma includes a reactive B-cell zone with secondary lymphoid follicles (black arrows demonstrate the germinal centers) (B), Hematoxylin and Eosin stain, 100×). The germinal centers comprise BCL-2 negative B-cells (C), BCL-2 immunostain, mouse monoclonal (clone 124), DAKO, dilution 1/40, 100×). The T-cell zone around the lymphoid follicles is quite prominent, as demonstrated by CD3 immunostain (D), CD3 immunostain, rabbit polyclonal, Zytomed, dilution 1/300, 100×). CD138 immunomarker highlights scattered, numerous plasma cells (E), CD138 immunostain, mouse monoclonal (clone MI15), DAKO, dilution 1/50, 100×). Immunohistochemistry against antigens of *Treponema pallidum* reveals few microorganisms with spiral coils, consistent with the morphology of spirochetes (F), *T. pallidum* immunostain, rabbit polyclonal, Abcam, dilution 1/100, 400×).

Gross examination revealed a tan-white tissue fragment, measuring 1.3 cm in maximum diameter (length) and a mildly solid cut surface. Microscopically, tissue segments of tonsillar tissue lined by mature squamous epithelium with focal ulceration and the presence of fibrinopurulent exudate were identified. The underlying stroma contained organized lymphoid tissue comprising of B-cell regions with secondary lymphoid follicles and B-cell lymphoma 2 (BCL-2)-negative germinal centers, as well as interfollicular T-cell zone and abundant scattered polytypic/polyclonal plasma cells, as demonstrated by the immunohistochemical markers for kappa (k) and lambda (l) light chains. No evidence of malignancy was observed. Considering the morphology of the lesion along with the rich polyclonal plasma cell infiltrate and the history of a penile ulcer, an immunohistochemical examination against *T. pallidum* was performed, revealing numerous microorganisms with morphology compatible with *T. pallidum* species (Figure 3). Following histopathological findings, serological tests confirmed syphilis, with positive Venereal Disease Research Laboratory (VDRL) (titer 1/2), *T. pallidum* Hemagglutination Assay (TPHA) (titer 1/640), and Fluorescent Treponemal Antibodies-Absorbed (FTA-ABS) (titer 1/20). Further serological testing for HIV, HBV, and hepatitis C virus (HCV) yielded negative results.

**Figure 4:**
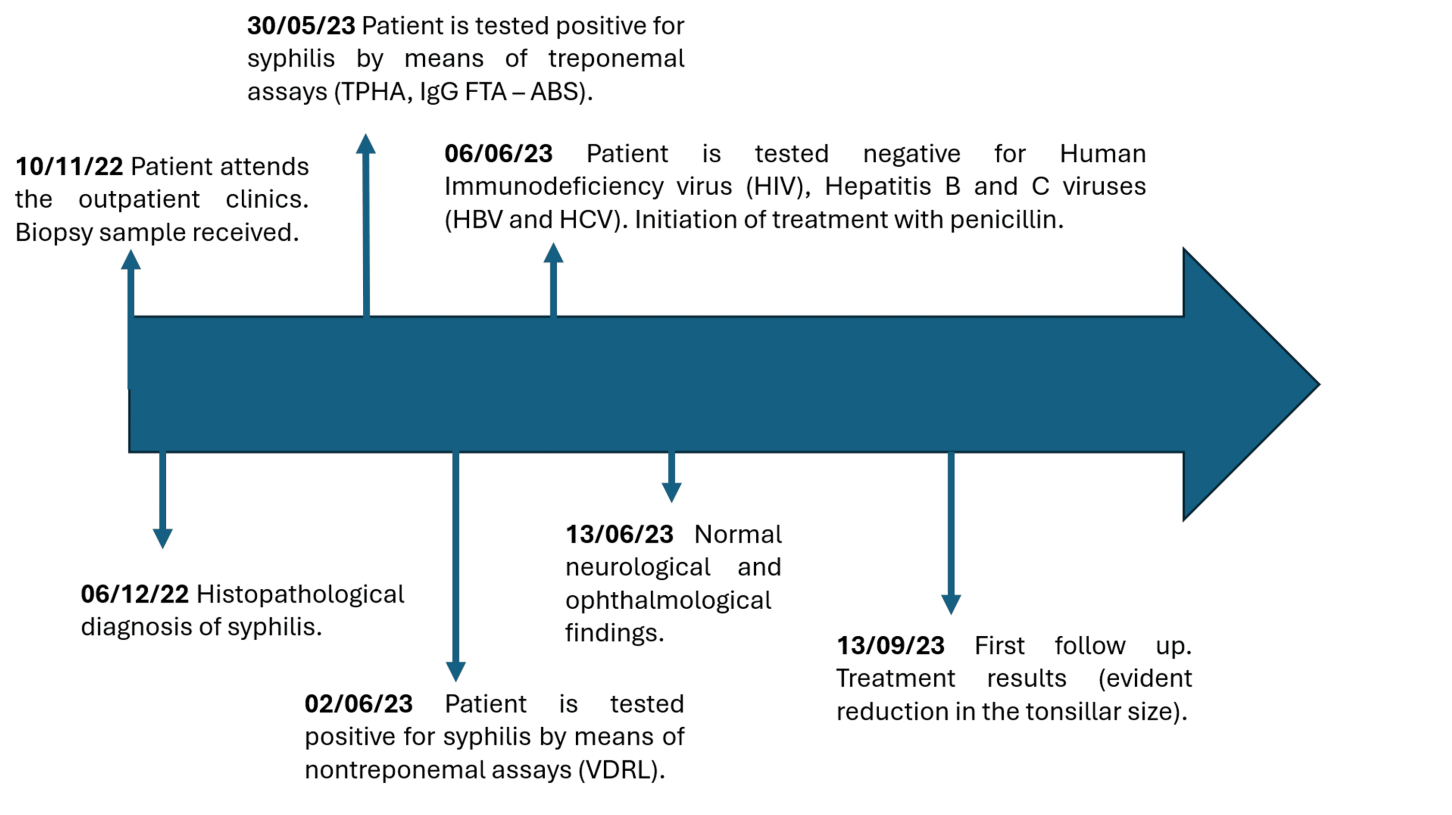
Timeline. Depiction of important dates in our case. TPHA: *T. pallidum* Hemagglutination Assay; FTA-ABS: Fluorescent Treponemal Antibodies-Absorbed

The patient was promptly initiated on benzathine penicillin G (IM single dose, 2.4 million units) to treat the underlying syphilis. He was closely monitored by dermatology and otolaryngology specialists. Follow-up assessments were planned to monitor treatment response and assess potential complications (Figure 1). A timeline of the clinical workup is included below (Figure 4). To summarize, the most distinguishing clinical features of our case were painless tonsillar swelling, lymphadenopathy, and a history of penile ulcer. This pattern recognition would help clinicians identify similar presentations and avoid misdiagnosis (e.g., tonsillar malignancy, chronic tonsillitis).

## Discussion

The literature on oropharyngeal syphilis provides valuable insights into its clinical presentation, diagnosis, and management. However, recognizing oropharyngeal syphilis as a potential manifestation of systemic infection remains critical [5]. This condition often presents with nonspecific symptoms such as sore throat, odynophagia, and cervical lymphadenopathy, which may mimic more common infections, such as bacterial tonsillitis or viral pharyngitis [6,7]. Our patient exhibited painless unilateral tonsillar swelling and cervical lymphadenopathy, an uncommon clinical presentation. Diagnosing oropharyngeal syphilis is particularly important in HIV-infected individuals, who may present with shallow ulcers, mucous patches, or macular lesions as early signs of secondary syphilis [8]. The variable presentation, combined with patient reluctance to disclose sexual history, can delay diagnosis, leading to inadequate treatment and disease progression [9].

The diagnosis of syphilis is largely based on serological tests. The traditional approach uses a nontreponemal assay for initial evaluation (e.g., VDRL, rapid plasma reagin/RPR test), followed by a treponemal assay (e.g., FTA-ABS test) for positive samples. An alternative approach, known as the reverse sequence algorithm, is preferred in cases of early or latent syphilis due to the lower sensitivity of nontreponemal tests in these stages [10]. Treponemal tests are more sensitive in early infection and once positive they remain reactive indefinitely. They cannot be used to monitor treatment response. Nontreponemal tests detect total antibodies against lipoidal antigens, which are released from damaged host cells and bacteria. They are quantitative and reported in titers. They are less sensitive in the very early and latent phases of the infection and are employed to monitor disease activity and response to treatment [10]. The serologic profile of our patient was consistent with secondary syphilis (positive for both treponemal and non-treponemal assays). A biopsy should only be considered for confirmatory reasons [6,10]. In our case, the suspicion of syphilis was raised by the pathologists and was then confirmed by treponemal assays (FTA-ABS, treponema pallidum particle agglutination (TPPA)).

The main histopathological characteristic of oropharyngeal syphilis is a dense plasmacytic/lymphocytic inflammatory infiltrate, which might acquire a perivascular or perineural distribution. Epithelial hyperplasia, as well as obliterative endarteritis, could also be observed. Sometimes, the presence of ulcerated lesions with fibrinopurulent exudate is noted. Lesions of tertiary syphilis may demonstrate chronic granulomatous reaction (“gummas”) [6]. The immune response in the upper aerodigestive tract can be either plasma cell-rich or lymphoma-like with activated large atypical lymphoid cells [11]. According to the above characteristics the differential diagnosis includes numerous entities, among which lymphomas. Of major concern are B-cell lymphomas [11], importantly extranodal marginal zone lymphoma of mucosa-associated lymphoid tissue, especially when monotypic plasmacytic differentiation occurs, and follicular lymphoma (in most cases CD10(+) and BCL-2 (+)). Our case exhibited BCL-2(-), CD10(+) germinal centers whereas the accompanying plasmacytic infiltrate was polytypic by immunohistochemistry for k and l light immunoglobin stains, thus excluding the possibility for these lymphomas. Given the dense plasmacytic infiltrate, IgG4-related disease must be ruled out [11]. However, in our patient, neither IgG4-positive cells nor the IgG4:IgG ratio were assessed. Mucosal ulcers associated with a polymorphous lymphoid infiltrate in head and neck lesions might be Epstein-Barr Virus (EBV) related, namely EBV-positive mucocutaneous ulcers [6]. The absence of atypical large cells positive for CD20 and CD30 along with no kind of immune suppression or dysregulation in the clinical history discouraged us from testing the sample for EBV with Epstein-Barr virus-encoded small ribonucleic acid (EBER) in-situ hybridization. Whenever granulomas are present the absence of fungal or mycobacterial infection must be excluded [11]. We did not observe the formation of granulomas microscopically, however, periodic acid-Schiff (PAS) and Grocott-Gomori’s methenamine silver (GMS) histochemical stains were performed to exclude fungal infection because of the co-existing neutrophilic infiltrate. The possibility of pseudoepitheliomatous hyperplasia may lastly be considered [12]. It is therefore of utmost importance for the pathologist to be informed by the clinicians whether there is suspicion of syphilis, so as to employ Silver stain or immunohistochemistry for *T. pallidum* in biopsy specimens as soon as possible for the detection of *Treponema* species.

Finally, prompt treatment is important to prevent complications and reduce the risk of transmitting the infection to others. Antibiotics, particularly penicillin, remain the gold standard for treating syphilis [13]. For patients with penicillin allergies, alternative regimens, such as doxycycline or ceftriaxone, have been explored with varying efficacy [14].

## Conclusions

Unilateral tonsillar swelling has diverse etiologies, and syphilis should be included in the differential diagnosis, even in atypical cases. Pathologists should consider syphilis, particularly when ulcerated lesions with abundant plasma cells are observed in the appropriate clinical context. Early recognition and prompt treatment of syphilis are essential to prevent disease progression and associated complications.
